# Factors associated with death in patients admitted with Ebola virus disease to Ebola Treatment Units in Guinea, Sierra Leone, and Liberia – December 2013 to March 2016

**DOI:** 10.12688/f1000research.149612.2

**Published:** 2025-03-03

**Authors:** Trokon Omarley Yeabah, Ibrahima Kaba, Gomathi Ramaswamy, Prabin Dahal, Alexandre Delamou, Benjamin T. Vonhm, Ralph W. Jetoh, Laura Merson, Adam C. Levine, Pryanka Relan, Anthony D. Harries, Ajay M.V. Kumar

**Affiliations:** 1 Department of Technical Services, National Public Health Institute of Liberia, Monrovia, Montserrado, 1000, Liberia; 2 African Center of Excellence for the Prevention and Control of Transmissible Diseases, University Gamal Abdel Nasser, Conakry, Guinea, 1017, Guinea; 3 All India Institute of Medical Sciences, Bibinagar, Hyderabad, 508126, India; 4 Infectious Diseases Data Observatory, Centre for Tropical Medicine & Global Health, University of Oxford, Headington, Oxfordshire, OX3 7LG, UK; 5 Centre national de formation et de recherche en santé rurale de Maferinyah, University Gamal Abdel Nasser, Forécariah, Conakry, 1017, Guinea; 6 ISARIC, Pandemic Sciences Institute, University of Oxford, Old Road Campus, Headington, Oxfordshire, OX3 7LG, UK; 7 Warren Alpert Medical School, Brown University, Providence, Rhode Island, 02903, USA; 8 Health Emergencies Programme, World Health Organization, Avenue Appia 20, Geneva, 1203, Switzerland; 9 Department of Clinical Research, Faculty of Infectious and Tropical Diseases, London School of Hygiene and Tropical Medicine, Keppel Street, London, WC1E 7HT, UK; 10 International Union Against Tuberculosis and Lung Disease, The Union, 2 Rue Jean Lantier, Paris, 75001, France; 11 Yenepoya Medical College, Yenepoya Deemed to be University, Deralakatte, Mangalore, 575018, India; 12 South-East Asia Office, The Union, C6, Qutub Institutional Area, New Delhi, 110016, India

**Keywords:** West Africa, Ebola, mortality, viral haemorrhagic fever, filovirus, SORT IT, operational research, pandemic preparedness

## Abstract

**Background:**

The 2013-2016 West African Ebola Virus Disease (EVD) outbreak resulted in 28,600 cases and 11,300 deaths officially reported to the World Health Organization. Previous studies investigating factors associated with death had conflicting findings, interventions showing promising outcomes had small sample sizes, studies were often single- or dual-country based and most focused on laboratory-confirmed EVD and not on clinically-suspected EVD. We used the Ebola data platform of the Infectious Disease Data Observatory (IDDO) to review individual patient records to assess factors associated with death, and particularly whether there were differences between laboratory-confirmed and clinically-suspected cases.

**Methods:**

This was a cohort study involving analysis of secondary data in the IDDO database. The study population included all patients classified as having either clinically-suspected or laboratory-confirmed EVD, admitted to 22 Ebola Treatment Units (ETU) in Guinea, Liberia and Sierra Leone between December 2013 and March 2016. Baseline characteristics and treatments were documented along with ETU exit outcomes. Factors associated with death were investigated by multivariable modified Poisson regression.

**Results:**

There were 14,163 patients, of whom 6,208 (43.8%) were laboratory-confirmed and 7,955 (56.2%) were clinically-suspected. Outcomes were not recorded in 2,889 (20.4%) patients. Of the 11,274 patients with known outcomes, 4,090 (36.3%) died: 2,956 (43.6%) with laboratory-confirmed EVD and 1,134 (18.8%) with clinically-suspected EVD. The strongest risk factor for death was confirmed disease status. Patients with laboratory-confirmed disease had 2.9 times higher risk of death compared to clinically-suspected patients, after adjusting for other co-variables. Other factors significantly associated with death included a higher risk for patients aged ≥60 years and a lower risk for patients in Sierra Leone.

**Conclusions:**

Although laboratory-confirmed patients admitted to ETUs fared worse than clinically-suspected patients, the latter still had a substantial risk of death and more attention needs to be paid to this group in future EVD outbreaks.

## Introduction

Ebola virus disease (EVD) is a severe, often fatal, zoonotic, filovirus illness that was documented for the first time in the Democratic Republic of Congo (formerly Zaire) in 1976. Since then, there have been several outbreaks, with the largest and deadliest outbreak occurring in West Africa (primarily Guinea, Sierra Leone, and Liberia) between 2013 and 2016, with approximately 28,600 cases and 11,310 deaths officially reported to the World Health Organization (WHO) ( WHO - emergencies). ^1^ The disease remains a public health threat due to its high case-fatality ratio and the potential for the virus to lie dormant in animal reservoirs and then re-emerge. Recent outbreaks in the Democratic Republic of Congo in 2017-2022, Guinea in 2021, and Uganda in 2022 illustrate this ongoing concern ( CDC - Ebola outbreaks). Despite recurring outbreaks, many aspects of EVD remain poorly understood. ^2^ There remains a need to further understand the relationship between the signs and symptoms, the spectrum of illness, and outcomes, as well as the influence of co-existing infections and environmental factors on disease course and outcomes. ^3^^,^^4^

The 2013-2016 West African EVD outbreak was associated with an overall case fatality ratio of 51% (95% CI, 46%-56%), pooled from 16 independent cohorts of over 6,000 patients. ^5^ Various cohort studies investigated risk factors associated with death in adults and children, both separately and together. Demographic factors such as age (elderly and young children) and male gender appear to be important factors associated with increased risk of death. ^6^^–^^9^ Clinical characteristics that include symptoms such as fever, diarrhoea, vomiting, dysphagia, cough, and dyspnoea, and physical signs such as skin rash, conjunctival injection, and haemorrhagic manifestations have been independently associated with high-case fatality in many studies, ^10^^–^^13^ although these associations have not been consistent. ^9^ Certain laboratory investigations such as hyponatraemia, hypokalaemia, hyperkalaemia, elevated liver enzymes, high serum creatinine, and high EVD viral load have been associated with high risk of death. ^7^^,^^14^ Co-infection with malaria appears to be a risk factor for death. ^15^ Finally, there are a few studies with small numbers of patients that have found various interventions beneficial in reducing case fatality: multivitamins or vitamin A given within 48 hours of admission, ^16^^,^^17^ antibiotics such as third-generation oral cephalosporins especially cefixime, ^17^ and use of empirical antimalarial treatment, especially artesunate-amodiaquine rather than artemether-lumefantrine. ^18^^,^^19^

While existing published studies have examined risk factors for mortality, several reasons justify the need for additional research in this area. First, there have been conflicting findings between studies, especially with respect to clinical characteristics associated with mortality. ^14^ Second, interventions that have shown promising outcomes, such as the use of certain antibiotics, multivitamins, and antimalarial drugs, were based on small sample sizes. Third, many of the previous studies included data from one or two countries, limiting their generalizability. Lastly, most previous studies focused on mortality in patients with laboratory-confirmed EVD, and there is limited information about clinical characteristics and outcomes in patients with clinically-suspected EVD. ^6^^,^^9^^,^^20^

The Infectious Disease Data Observatory (IDDO) hosts an Ebola Data Platform (EDP), the first multi-country repository for clinical, epidemiological, and laboratory data, on patients with suspected EVD. Data from over 14,000 individual patient records collected during the 2013–16 West African Ebola outbreak have been deposited with the aim of reducing the impact of EVD by generating new evidence to improve outbreak response and patient care. This resource allows the unique possibility of examining a large dataset, with data combined from three countries -- Guinea, Liberia, and Sierra Leone - to generate further evidence on risk factors for mortality and on interventions that can reduce mortality in patients with both clinically-suspected and laboratory-confirmed EVD.

The aim of this study was to assess factors associated with mortality during admission to Ebola Treatment Units (ETU) among patients with clinically-suspected and laboratory-confirmed EVD admitted to 22 ETUs in Guinea, Liberia, and Sierra Leone between December 2013 and March 2016. The specific objectives were to: i) describe the baseline socio-demographic and clinical characteristics, laboratory investigations, and treatments received; ii) determine the ETU exit outcomes, including the proportion who died during hospitalization and the median time from onset of symptoms to admission and admission to death; and iii) assess the baseline socio-demographic and clinical characteristics, and treatments that were associated with risk of death during hospitalization.

## Methods

### Study design

This was a cohort study involving analysis of secondary data collected from Guinea, Liberia, and Sierra Leone.

### Setting

***General setting***

**Figure 1.**
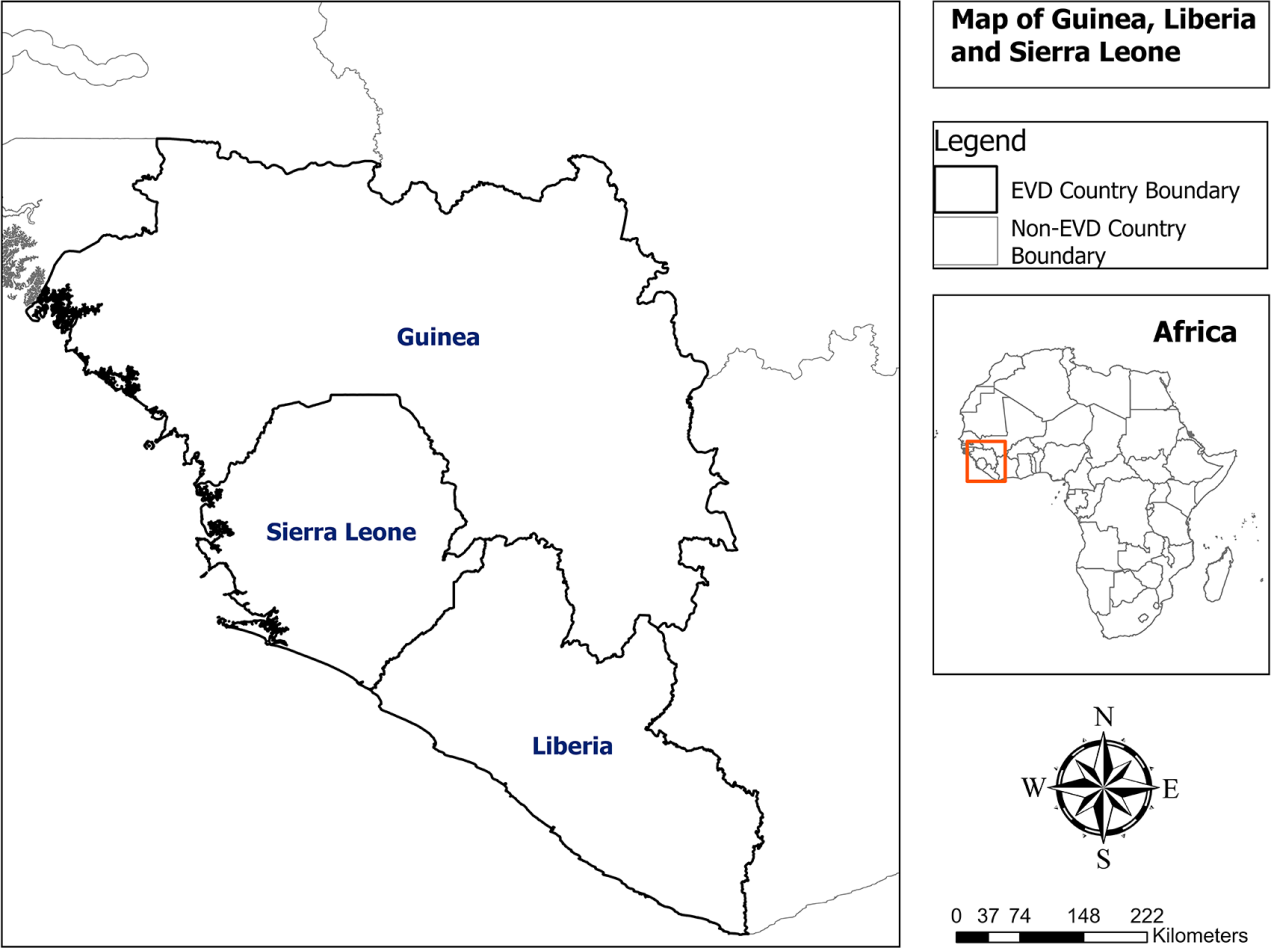
Map of Guinea, Liberia and Sierra Leone.

Guinea, Liberia, and Sierra Leone are Member States of the Mano River Union Basin located in West Africa, with an estimated population of 23.5 million inhabitants in 2015 ( Figure 1). ^21^

Guinea is a country bordered by Guinea-Bissau to the northwest, Senegal to the north, Mali to the northeast, Côte d’Ivoire to the southeast, and Liberia and Sierra Leone to the south, with an estimated population of 11.6 million inhabitants. ^21^ It has 8 administrative regions with 38 health districts and 936 health facilities. ^22^

Liberia is a country bordered by Sierra Leone to the northwest, Guinea to the north, Ivory Coast to the east, and the Atlantic Ocean to the south and southwest, with an estimated population of 4.6 million inhabitants. ^21^ It has 15 counties with 98 health districts and 978 health facilities. ^23^

Sierra Leone is a country bordered by Liberia to the southeast and Guinea to the northern half, with an estimated population of 7.3 million inhabitants. ^21^ It has four provinces with 13 health districts and 1,280 health facilities. ^24^

***Specific setting***

Across the three countries, ETUs were set up in hospitals and other external designated sites by Ministries of Health and Non-Governmental Organizations (NGOs) for the case management of patients with suspected and confirmed EVD. These ETUs were subdivided into sections for processing patients that included triage, admission, and treatment wards. Patients who arrived at the ETUs were triaged, and demographic and clinical characteristics, medical history, and environmental risk factors such as funeral attendance were collected.

A polymerase chain reaction (PCR) test was then carried out for the determination of EVD. Patients were then transferred to separate treatment unit wards depending on whether they had laboratory-confirmed EVD or clinically-suspected EVD. For the purpose of this study, laboratory-confirmed EVD was defined as “confirmed” disease status assigned by the original study investigators when the data was submitted to the IDDO platform. In the absence of investigator assigned confirmed status, laboratory-confirmed EVD was based on a positive polymerase chain reaction (PCR) test or a cycle threshold of 36.1 or less, obtained within the first three days of reporting to the health facility. Clinically-suspected EVD was based on a negative/indeterminate PCR test and/or a cycle threshold greater than 36.1 or no laboratory information available in the dataset.

In ETU wards, additional data were collected routinely on clinical care and follow-up, including laboratory and epidemiological investigations. These data were often used for patient management and occasionally for observational or interventional research. Clinical trials on EVD therapeutics such as monoclonal antibody therapies and convalescent plasma were conducted at some ETUs. These sites had local and international medical doctors, nurses, laboratory technicians, epidemiologists, logisticians, and other support personnel. Data were collected on either paper-based or electronic forms using variables selected by the organization managing the respective clinical treatment centres and those undertaking studies. The data were submitted to the EDP by the organization responsible for primary data collection under the authority of the responsible Ministry of Health or National Public Health Institute.

The EDP team aggregated and standardized disparate datasets from the many organizations that collected individual patient-level data as a part of the care provided in ETUs. The data were hosted on the University of Oxford data repository server. The curation of the data was done using a Clinical Data Interchange Standards Consortium (CDISC) compliant model and the standardised data are stored across several study data tabulation model (SDTM) domains.

Table 1 shows the total number of ETUs ^25^ in each country, the number and proportion included in the study ( IDDO - Ebola), the number of EVD cases reported to WHO, ^26^^,^^27^ and the number and proportion included in the study.

**Table 1.** Description of Ebola Treatment Units (ETUs) and Ebola Virus Disease (EVD) cases by country.

Country	No. ETUs	No. (%) ETUs in IDDO database	No. EVD cases reported to WHO	No. (%) admitted cases to ETU in IDDO database
Guinea	10	5 (50)	3,814	5,448 (143)
Liberia	25	4 (16)	10,678	3,623 (34)
Sierra Leone	22	13 (59)	14,124	5,092 (36)
Total	57	22 (39)	28,616	14,163 (50)

IDDO=Infectious Disease Data Observatory; WHO=World Health Organization.

### Study population

The study population included all patients classified as having either clinically-suspected or laboratory-confirmed EVD who were admitted at the 22 ETUs in Guinea, Liberia and Sierra Leone between December 2013 and March 2016 and who were captured in the IDDO database.

### Data source and data variables

The data variables were extracted from the Ebola Database Platform (EDP) database (using the variable name from the respective SDTM Domains) according to the specific study objectives. The key exposure variable was ‘confirmed disease status’ (laboratory-confirmed or clinically-suspected). Socio-demographic characteristics included: country, age, sex, pregnancy status, healthcare worker, contact with an individual suspected of EVD infection, visit to a traditional healer and funeral attendance. Baseline clinical features included: general, gastrointestinal, respiratory, neurological, and ocular symptoms; a variety of physical signs such as fever, rash; vital signs such as temperature, pulse rate, respiratory rate and blood pressure; and investigations for diagnosis of malaria. The signs and symptoms data were extracted only if they were indicated as pre-specified variables (i.e. those variables that were actively solicited in the case report form). Baseline was defined as any clinical feature present at least once between the date of reporting to the health facility and 21 days before reporting to the health facility. Availability of laboratory tests varied between patients and ETUs and included serum sodium, serum potassium, blood urea, serum creatine and liver function tests such as aspartate transaminase and alanine transaminase. Treatments received included: multivitamins, antimalarial drugs, antibiotics, intravenous fluids and parenteral nutrition. ETU exit outcomes included: discharge, death, and other (withdrawn from clinical trial, transferred, lost to follow-up, still in hospital or unknown). We also measured number of days from symptom onset to ETU admission and number of days from ETU admission to discharge or death.

### Data analysis

The variables of interest were extracted from the EDP database, cleaned, duplicates were removed, data elements were recoded including missing data and a standardised dataset was formulated to allow analyses to proceed. These data were then imported to Stata Statistical Software (Release 18.0, StataCorp. LLC, College Station, Texas, USA) and R Studio (version 2023.06.1 Build 524, IDE PBC, Boston, MA, USA) for further analysis. While Stata is a proprietary software, all the analyses presented in the manuscript can be replicated using R Studio, which is an open access software. Categorical variables such as socio-demographic baseline characteristics and ETU exit outcomes were summarised using frequencies and proportions while continuous variables were summarised using mean (and standard deviations) or median (and interquartile range), as appropriate.

Differences in proportion of deaths across the various sub-groups (based on laboratory-confirmed and clinically-suspected EVD, demographic and clinical characteristics and treatments) were assessed for statistical significance using the Chi-squared test or Fisher’s exact test, as appropriate. The strength of associations was measured using risk ratios (RR) and 95% confidence intervals (CI), with the level of significance set at *P*-Value <0.05. Univariable and multivariable predictors associated with death were assessed using modified Poisson regression with robust variance estimation using *rqlm* package in R Studio software. ^28^ Variables found to be associated with hospital death on unadjusted analysis were further assessed in multivariable analysis, and adjusted RRs with 95% CI were calculated. Clinical signs and symptoms were not included in the multivariable model as they were highly correlated with each other and with the confirmed disease status. Patients who received treatments (such as antibiotics, antimalarials, IV fluids and multivitamins) often received them in combination and hence it was not possible to assess the effects of each of the treatments. A composite variable ‘receiving any treatment’ was created and used in the multivariable analysis.

### Ethics

The hosting of data and access to the EDP were approved by the Oxford Tropical Research Ethics Committee, UK, in 2018 and by the national ethics committees in each contributing country. The Guinea National Committee for Health Research Ethics (2018), the Sierra Leone Ethics and Scientific Review Committee (2018) and the Liberia National Research Ethics Board (2018) all approved the activities of the EDP. For the current study, approval was obtained from the Ethics Advisory Group (EAG) of the International Union against Tuberculosis and Lung Disease, Paris, France (date of approval 08/09/2023; EAG approval number 19/23). Approval was also obtained from the Liberia National Research Ethics Board (date of approval 27/09/2023; approval number 23-09-389). No identifiable data were included in the analysis. The study used anonymised secondary collected data and as such no informed consent was needed from patients.

## Results

There were 14,163 patients admitted to the study ETUs in Guinea, Liberia, and Sierra Leone during the study period. Of these, 6,208 (43.8%) were laboratory-confirmed and the remainder (n=7,955, 56.2%) were clinically-suspected. The proportion of laboratory-confirmed patients varied across the countries: 43.6% in Guinea, 50.0% in Liberia and 39.7% in Sierra Leone.

### Baseline socio-demographic characteristics

The distribution of socio-demographic characteristics of patients disaggregated by laboratory confirmation status is shown in Table 2. Overall, 51.8% of the patients were male – this was higher among clinically-suspected compared to laboratory-confirmed patients. Among 6,715 females, 195 (2.9%) were pregnant. The age distribution across the two groups was similar, barring a marginally higher proportion of under-five children in clinically-suspected patients. Nearly one in 10 patients were healthcare workers; this proportion was higher among laboratory-confirmed (15.0%) compared to clinically-suspected (7.0%) patients. 42.8% of patients had a history of contact with an EVD suspect, and 17.8% had a history of funeral attendance – these proportions were higher among laboratory-confirmed patients compared to the clinically-suspected cases. A higher proportion of clinically-suspected patients had visited a traditional healer compared to laboratory-confirmed patients (6.8% vs 3.3%).

**Table 2.** Sociodemographic characteristics of patients with laboratory-confirmed and clinically-suspected EVD in Ebola Treatment Units in Guinea, Liberia, and Sierra Leone - December 2013 to March 2016.

Characteristics		N (%)	Clinically- suspected	Laboratory-confirmed	*P*-value
**Total**		**14,163**	**7,955**	**6,208**	
**Socio-demographic **					
Country	Guinea	5,448 (38.5)	3,075 (38.7)	2,373 (38.2)	
	Liberia	3,623 (25.6)	1,811 (22.8)	1,812 (29.2)	<0.001
	Sierra Leone	5,092 (36.0)	3,069 (38.6)	2,023 (32.6)	
Sex (n=13,933)	Female	6,715 (48.2)	3,554 (45.2)	3,161 (52.1)	
	Male	7,218 (51.8)	4,309 (54.8)	2,909 (47.9)	<0.001
	Missing	230	92	138	
Age in years (n=13,830)	≤5	1,275 (9.2)	852 (10.9)	423 (7.0)	
	6 – 18	2,301 (16.6)	1,147 (14.7)	1,154 (19.1)	
	19 – 39	6,242 (45.1)	3,544 (45.5)	2,698 (44.7)	<0.001
	40 – 59	2,949 (21.3)	1,602 (20.6)	1,347 (22.3)	
	≥60	1,063 (7.7)	651 (8.4)	412 (6.8)	
	Missing	333	159	174	
Pregnancy (n=6,715)	Yes	195 (2.9)	122 (3.4)	73 (2.3)	
	No	6,520 (97.1)	3,432 (96.6)	3,088 (97.7)	0.006
Malaria (n=2,145)	Negative	1,491 (69.5)	1,018 (65.1)	473 (81.4)	<0.001
	Positive	654 (30.5)	546 (34.9)	108 (18.6)	
	Missing	12,018	6,391	5,627	
Healthcare Worker (n=3,869)	Yes	373 (9.6)	184 (7.0)	189 (15.0)	
	No	3,496 (90.4)	2,428 (93.0)	1,068 (85.0)	<0.001
	Missing	10,294	5,343	4,951	
Contact with suspect (n=4,831)	Yes	2,068 (42.8)	757 (26.8)	1,311 (65.4)	<0.001
	No	2,763 (57.2)	2,069 (73.2)	694 (34.6)	
	Missing	9,332	5,129	4,203	
Visited Traditional Healer (n=3,125)	Yes	175 (5.6)	139 (6.8)	36 (3.3)	<0.001
	No	2,950 (94.4)	1,891 (93.2)	1,059 (96.7)	
	Missing	11,038	5,925	5,113	
Funeral attendance (n=4,498)	Yes	800 (17.8)	274 (9.9)	526 (30.4)	<0.001
	No	3,698 (82.2)	2,492 (90.1)	1,206 (69.6)	
	Missing	9,665	5,189	4,476	

Column percentages are calculated after excluding missing data; EVD – Ebola Virus Disease.

### Clinical characteristics

The pre-specified clinical symptoms and signs presented by the patients at the time of admission are shown in Table 3. The most common symptoms were fever, fatigue/lethargy, myalgia/arthralgia, anorexia/dehydration, diarrhoea, nausea/vomiting, abdominal pain and neurological symptoms (which included headache, seizures/convulsions, agitation, disorientation, coma/unconsciousness, confusion, dizziness). Other symptoms included chest pain, difficulty breathing, difficulty swallowing, sore throat, hiccups and bleeding. The following symptoms were proportionately higher among the laboratory-confirmed cases: fatigue, nausea/vomiting, diarrhoea, anorexia & dehydration, fever, myalgia/arthralgia, neurological symptoms, hiccups, difficulty swallowing, sore throat, and ocular complaints. The proportion of patients with abdominal pain and bleeding (internal and external) was similar in laboratory-confirmed patients and clinically-suspected patients.

**Table 3.** Baseline clinical characteristics of patients with laboratory-confirmed and clinically-suspected EVD in Ebola Treatment Units in Guinea, Liberia, and Sierra Leone - December 2013 to March 2016.

Characteristics		N (%)	Clinically- suspected	Laboratory-confirmed	* P*-value
**Total**		**14,163**	**7,955**	**6,208**	
Fatigue lethargy pallor (n=10,369)	Yes	8,115 (78.3)	3,794 (72.2)	4,321 (84.5)	
	No	2,254 (21.7)	1,464 (27.8)	790 (15.5)	<0.001
	Missing	3,794	2,697	1,097	
Nausea/Vomiting (n=10,244)	Yes	5,755 (56.2)	2,638 (50.8)	3,117 (61.7)	
	No	4,489 (43.8)	2,552 (49.2)	1,937 (38.3)	<0.001
	Missing	3,919	2,765	1,154	
Diarrhoea (n=10,129)	Yes	5,034 (49.7)	1,992 (39.1)	3,042 (60.4)	
	No	5,095 (50.3)	3,102 (60.9)	1,993 (39.6)	<0.001
	Missing	4,034	2,861	1,173	
Neurological symptoms (n=10,079) ^#^	Yes	5,602 (55.6)	2,961 (57.5)	2,641 (53.6)	
	No	4,477 (44.4)	2,189 (42.5)	2,288 (46.4)	<0.001
	Missing	4,084	2,805	1,279	
Anorexia & dehydration (n=10,015)	Yes	6,965 (69.5)	3,345 (65.1)	3,620 (74.2)	<0.001
	No	3,050 (30.5)	1,791 (34.9)	1,259 (25.8)	
	Missing	4,148	2,819	1,329	
Fever (n=9,962)	Yes	7,734 (77.6)	3,955 (75.7)	3,779 (79.8)	
	No	2,228 (22.4)	1,270 (24.3)	958 (20.2)	<0.001
	Missing	4,201	2,730	1,471	
Difficulty breathing (n=8,659)	Yes	1,934 (22.3)	1,204 (27.7)	730 (16.9)	
	No	6,725 (77.7)	3,144 (72.3)	3,581 (83.1)	<0.001
	Missing	5,504	3,607	1,897	
Abdominal pain (n=8,570)	Yes	4,589 (53.5)	2,435 (53.0)	2,154 (54.1)	0.310
	No	3,981 (46.5)	2,156 (47.0)	1,825 (45.9)	
	Missing	5,593	3,364	2,229	
Bleeding - Internal and external (n=9,457)	Yes	1,106 (11.7)	562 (11.4)	544 (12.0)	
	No	8,351 (88.3)	4,366 (88.6)	3,985 (88.0)	0.359
	Missing	4,706	3,027	1,679	
Myalgia/arthralgia (n=8,671)	Yes	5,132 (59.2)	2,661 (57.4)	2,471 (61.2)	
	No	3,539 (40.8)	1,972 (42.6)	1,567 (38.8)	<0.001
	Missing	5,492	3,322	2,170	
Ocular complaints (n=8,638)*	Yes	1,827 (21.2)	656 (15.0)	1,171 (27.5)	
	No	6,811 (78.8)	3,717 (85.0)	3,094 (72.5)	<0.001
	Missing	5,525	3,582	1,943	
Hiccups (n=8,386)	Yes	1,132 (13.5)	541 (12.1)	591 (15.1)	
	No	7,254 (86.5)	3,941 (87.9)	3,313 (84.9)	<0.001
	Missing	5,777	3,473	2,304	
Difficulty in swallowing (n=7,385)	Yes	1,895 (25.7)	924 (23.6)	971 (28.0)	
	No	5,490 (74.3)	2,991 (76.4)	2,499 (72.0)	<0.001
	Missing	6,778	4,040	2,738	
Rash (n=5,409)	Yes	265 (4.9)	147 (6.6)	118 (3.7)	
	No	5,144 (95.1)	2,091 (93.4)	3,053 (96.3)	<0.001
	Missing	8,754	5,717	3,037	
Chest pain (n=5,015)	Yes	1,946 (38.8)	741 (42.0)	1,205 (37.1)	
	No	3,069 (61.2)	1,025 (58.0)	2,044 (62.9)	0.001
	Missing	9,148	6,189	2,959	
Sore throat (n=4,222)	Yes	1,106 (26.2)	376 (22.2)	730 (28.9)	
	No	3,116 (73.8)	1,318 (77.8)	1,798 (71.1)	<0.001
	Missing	9,941	6,261	3,680	

Column percentages are calculated after excluding missing data; EVD – Ebola Virus Disease; ^#^ Neurological symptoms included headache, seizures/convulsions, agitation, disorientation, coma/unconscious confusion, dizziness, some CNS symptoms ^*^Ocular symptoms included: Conjunctivitis, red eye, conjunctivitis (pink eye), conjunctival injection, red eyes, complaints of eye pain before presentation, eye pain, ocular pain, pain behind eye, sensitivity to light, pain in eyes, photophobia, sensation of foreign body, blurred vision pain both eyes, blurry vision, change in vision, eye problem, loss of vision, vision/ocular problem.

The following symptoms and signs occurred less among laboratory-confirmed cases compared to clinically-suspected cases: difficulty breathing (16.9% vs 27.7%) and rash (3.7% vs 6.6%).

Altogether, only 2,145 (15.1%) EVD patients had malaria laboratory results available. Of these, 654 (30.5%) had a positive diagnosis, this being more common in those with clinically-suspected EVD (34.9%, 546/1,564) compared to those with laboratory-confirmed EVD (18.6%, 108/581).

Vital signs (respiratory rate, pulse rate, blood pressure etc.) and laboratory parameters such as serum electrolytes and renal or liver function tests were documented in <1% of the patients and hence were not included in the analysis (data not shown).

### Treatments

In patients with documented treatment information, the common treatments received included multivitamins, antimalarials, antibiotics, and intravenous fluids ( Table 4). The proportions of patients who received cephalosporins and IV fluids were similar between laboratory-confirmed and clinically-suspected patients. The proportion of patients who received multivitamins and antimalarial drugs were slightly higher among clinically-suspected patients.

**Table 4.** Treatments received at baseline in laboratory-confirmed and clinically-suspected patients with EVD in Ebola Treatment Units in Guinea, Liberia, and Sierra Leone - December 2013 to March 2016.

Characteristics		N (%)	Clinically-suspected	Laboratory-confirmed	*P*-Value
**Total**		**14,163**	**7,955**	**6,208**	
Multivitamins (n=2,949)	Yes	2,244 (76.1)	1,094 (80.9)	1,150 (72.0)	
	No	705 (23.9)	258 (19.1)	447 (28.0)	<0.001
	Missing	11,214	6,603	4,611	
Antimalarial Drugs (n=3,480)	Yes	3,061 (88.0)	1,552 (90.9)	1,509 (85.2)	
	No	419 (12.0)	156 (9.1)	263 (14.8)	<0.001
	Missing	10,683	6,247	4,436	
Antibiotics (Others) (n=2,583)	Yes	154 (6.0)	50 (3.1)	104 (10.9)	
	No	2,429 (94.0)	1,583 (96.9)	846 (89.1)	<0.001
	Missing	11,580	6,322	5,258	
Antibiotics (Cephalosporins) (n=3,417)	Yes	2,906 (85.0)	1,439 (85.0)	1,467 (85.1)	
	No	511 (15.0)	254 (15.0)	257 (14.9)	0.937
	Missing	10,746	6,262	4,484	
Intravenous Fluids (n=2,785)	Yes	865 (31.1)	504 (30.8)	361 (31.4)	
	No	1,920 (68.9)	1,131 (69.2)	789 (68.6)	0.751
	Missing	11,378	6,320	5,058	
Peripheral Parenteral Nutrition (n=421)	Yes	2 (0.5)	0 (0.0)	2 (0.5)	
	No	419 (99.5)	12 (100.0)	407 (99.5)	0.808
	Missing	13,742	7,943	5,799	
Received any treatment (n=3,667) ^a^	Yes	3,380 (92.2)	1,670 (95.5)	1,747 (93.6)	
	No	287 (7.8)	78 (4.5)	120 (6.4)	0.009
	Missing	10,496	6,207	4,341	

Column percentages are calculated after excluding missing data; EVD – Ebola Virus Disease; ^a^Any treatment with one or more of the following: antimalarials, any antibiotic, vitamins or multivitamins, nutritional intervention or intravenous fluids.

### Outcomes

Overall, 50.7% (7,184/14,163) of the patients were discharged, 28.9% (4,090/14,163) of patients died and in the remaining 20.4% (2,889/14,163), the outcome was unknown (which included withdrawn from a clinical trial, transferred, lost to follow-up, still in hospital or unknown) ( Table 5). Death was substantially higher in laboratory-confirmed patients as compared to clinically-suspected patients (43.6% vs 18.8% among patients with known outcomes). The median duration from onset of symptoms to admission in the ETUs was 3 days (similar in both clinically-suspected and laboratory-confirmed patients). The median duration from admission to death was 4 days – this was higher at 4 days in laboratory-confirmed patients compared to 3 days in clinically-suspected patients. The median duration from admission to discharge was 4 days – this was higher at 13 days in laboratory-confirmed patients compared to 3 days in clinically-suspected patients.

**Table 5.** Outcome of laboratory-confirmed and clinically-suspected patients with EVD in Ebola Treatment Units in Guinea, Liberia, and Sierra Leone - December 2013 to March 2016.

Variables		N (%)	Clinically-suspected	Laboratory-confirmed	*P*-Value
**Total**		**14,163**	**7,955**	**6,208**	
**Characteristics**					
Outcome	Death	4,090 (36.3)	1,134 (18.8)	2,956 (43.6)	
	Discharged	7,184 (63.7)	4,897 (81.2)	2,287 (56.4)	<0.001
	Unknown	2,889	1,924	965	
Duration from symptom onset to admission (n=10,740)	Median [IQR] of days	3.0 [2.0, 6.0]	3.0 [1.0, 6.0]	4.0 [2.0, 7.0]	
Duration from admission to discharge/death (n=13,494)	Median [IQR] of days	4.0 [2.0, 8.0]	3.0 [2.0, 4.0]	7.0 [4.0, 13.0]	
Duration from admission to death (n=4,090)	Median [IQR] of days	4.0 [2.0, 6.0]	3.0 [1.0, 5.0]	4.0 [3.0, 7.0]	
Duration from admission to discharge (n=7,184)	Median [IQR] of days	4.0 [3.0, 11.0]	3.0 [2.0, 4.0]	13.0 [10.0, 16.0]	

Column percentages are calculated after excluding missing data; IQR=interquartile range.

### Factors associated with death in patients with EVD

Factors associated with death are shown in Table 6, the denominator for this analysis being patients with known outcome. Overall, a total of 11,274 patients had their outcome recorded as death or discharged and of these, 4,090 (36.3%) died. In multivariable analysis, the factors significantly associated with death included confirmed disease status, age and country. The strongest risk factor was laboratory-confirmed disease status. Patients with laboratory-confirmed disease had 2.9 times higher risk of death compared to clinically-suspected patients, after adjusting for other co-variables. Patients aged 60 years and above had a significantly higher risk of death compared to that in ≤5-year-old children, whereas those aged 6 – 39 years had a significantly lower risk of death compared with children aged 5 years or younger. Among the countries, Sierra Leone had the lowest risk of death compared to Guinea and Liberia.

**Table 6.** Risk factors for mortality among patients with EVD in Ebola Treatment Units in Guinea, Liberia, and Sierra Leone - December 2013 to March 2016 *(N=11,274 for whom outcome was known).*

Variables	Number of patients	Number (%) of deaths		RR [95% CI] ^a^	*P*-Value	aRR [95% CI] ^a^	*P*-Value
**Total**	**11,274**	**4,090**	**(36.3)**				
**Age-group in years**							
≤5	977	366	(37.5)	Reference	-	-	-
6-18	1,888	548	(29.0)	0.77 [0.69-0.86]	<0.001	0.65 [0.57-0.74]	<0.001
19-39	4,944	1,579	(31.9)	0.86 [0.78-0.94]	0.001	0.77 [0.69-0.86]	<0.001
40-59	2,345	994	(42.4)	1.13 [1.03-1.24]	0.010	0.97 [0.86-1.10]	0.641
≥60	855	415	(48.5)	1.29 [1.16-1.44]	<0.001	1.17 [1.02-1.35]	0.027
Unknown	265	188	(70.9)	1.89 [1.69-2.12]	<0.001	1.21 [0.97-1.52]	0.092
**Sex**							
Female	5,345	1,944	(36.4)	Reference	-	-	-
Male	5,733	1,991	(34.7)	0.96 [0.91-1.00]	0.071	1.04 [0.98-1.11]	0.173
Unknown	196	155	(79.1)	2.17 [2.01-2.36]	<0.001	1.58 [1.25-1.98]	<0.001
**Country**							
Guinea	3,452	1,444	(41.8)	Reference	-	-	-
Liberia	2,943	1,336	(45.4)	1.09 [1.03-1.15]	0.004	1.01 [0.94-1.09]	0.789
Sierra Leone	4,879	1,310	(26.9)	0.64 [0.60-0.68]	<0.001	0.67 [0.62-0.73]	<0.001
**Confirmed disease status** ^b^							
Clinically-suspected	6,031	1,134	(18.8)	Reference	-	-	-
Laboratory-confirmed	5,243	2,956	(56.4)	2.99 [2.83-3.17]	<0.001	2.93 [2.73-3.15]	<0.001
**Received any treatment** ^c^							
No	269	120	(44.6)	Reference	-	-	-
Yes	3,251	1,064	(32.7)	0.73 [0.64-0.85]	<0.001	0.93 [0.77-1.12]	0.450
Unknown	7,754	2,906	(37.5)	0.84 [0.73-0.96]	0.012	1.22 [1.01-1.47]	0.037
**Healthcare worker**							
No	2,046	410	(20.0)	Reference	-	-	-
Yes	230	63	(27.3)	1.36 [1.08-1.71]	0.007	0.91 [0.69-1.19]	0.481
Unknown	8,998	3,617	(40.2)	2.00 [1.83-2.19]	<0.001	1.16 [1.04-1.30]	0.006

Percentages in parenthesis are row percentages ^a^ RR=Unadjusted risk ratio; aRR=adjusted risk ratio; CI=confidence intervals estimated using modified Poisson regression with sandwich variance estimator.

^b^ The definition of confirmed disease status is outlined in the methodology section.

^c^ Any treatment with one or more of the following: antimalarials, any antibiotic, vitamins or multivitamins, nutritional intervention or intravenous fluids.

## Discussion

This analysis used the largest available Ebola clinical database to explore factors associated with death in laboratory-confirmed and clinically-suspected EVD cases in the three West African countries of Guinea, Liberia and Sierra Leone. The key finding was that almost half of the patients with laboratory-confirmed EVD died which was almost three times higher than in patients with clinically-suspected EVD, although a higher proportion of clinically-suspected cases had unknown exit outcomes which may have masked additional deaths.

There were other important findings. There were some baseline differences between laboratory-confirmed and clinically-suspected EVD in terms of characteristics and treatments given. In particular, those with clinically-suspected EVD had a higher proportion of males and individuals who had recently visited a traditional healer, and a lower proportion of patients who came into contact with a suspected patient and funeral attendance. Symptomatology and physical signs in general were less prevalent in those with clinically-suspected EVD compared with laboratory-confirmed EVD. A positive diagnosis of malaria was more common in those with clinically-suspected EVD, although in over 80% of patients the malaria status was unknown. The time from symptom onset to admission was similar in both groups, but the median time from admission to death or discharge was higher in those with laboratory-confirmed EVD compared with clinically-suspected EVD. Finally, on adjusted analysis, the confirmed disease status (laboratory-confirmed or clinically-suspected) was the strongest risk factor for death. In the multivariable analysis, older age (≥ 60 years) was associated with increased risk of death while being treated in Sierra Leone was associated with a decreased risk of death.

These study findings are important for several reasons. First, they show that clinically- suspected EVD patients still have an appreciable in-hospital mortality and thus justify the need for better on-going care and support. We defined clinically-suspected EVD on the basis of negative PCR tests up to day 3 of inpatient admission, but our datasets showed that patients could become PCR-positive from day 4 onwards. On-going repeat PCR testing in this group of patients is necessary, not only to improve confirmatory diagnoses for reporting and epidemiological purposes but also to further direct care and treatment.

Second, the findings support previous studies that show old age is a risk factor for death. ^6^^,^^7^^,^^9^^,^^29^ Older people with EVD thus need to be prioritised for ETU admission and targeted for appropriate care and support.

Third, it was discouraging, to see the lack of documentation of malaria testing. West Africa is endemic for this parasitic infection, and in more than 30% of the small number of patients tested there was a positive malaria diagnosis. Malaria is common and is a risk factor for death in EVD ^15^ and empirical antimalarial treatment may reduce case fatality. ^18^^,^^19^ Empiric treatment with antibiotics is frequently used as a supportive care component in the clinical management of EVD, the rationale being to mitigate the potential risk of secondary bacterial infection and gram-negative bacteraemia that arises during EVD. Oral cephalosporins may reduce the case fatality, ^17^ and should be explored further as treatment in patients ill with EVD.

There were several strengths to the study. There were large numbers of patients distributed between the three countries, giving enough power to test associations of baseline characteristics and treatments with exit outcomes. The conduct and reporting of the study adhered to STROBE (Strengthening the Reporting of Observational Studies in Epidemiology) guidelines. ^30^

There were some limitations. First, data on vital signs and laboratory investigations were only documented in a limited number of patients and we therefore could not investigate these as potential factors for mortality. Furthermore, there were large numbers of missing data for clinical characteristics and treatment. Second, we did not explore the impact of treatment as it was impossible to unravel the individual effects of vitamins, antibiotics and antimalarial treatment on EVD outcomes, as patients usually received a combination of these treatments at the time of ETU admission. Third, nearly one fifth of patients had unknown exit outcomes, which reduced the reliability and precision around analysis of risk factors associated with death. Previous investigations have highlighted that case fatality estimates can be substantially affected by these unknown outcomes and this remains a major limitation of the analysis. ^31^ It should also be noted that the findings on factors associated with death presented in our study should be interpreted solely as statistical associations which can be used for hypothesis generation; causal postulations remained beyond the scope of the current work. Fourth, we used a cycle threshold of 36.1 or less to define laboratory-confirmed EVD, while in other studies in West Africa and the Democratic Republic of Congo a cycle threshold >40 was considered negative when assessing various machine learning models to predict survival in children with suspected EVD. ^32^ Different results might therefore be obtained depending on how that cycle threshold is set. Fifth, we do not know the reasons for the discrepancy between the 5448 EVD cases in Guinea in the IDDO database and only 3814 EVD cases in Guinea reported to WHO. We suspect that cases reported to WHO included just those with confirmed EVD and those with suspected EVD were not reported, although we have no firm evidence to support this. Finally, extracting data from the large IDDO database was technically difficult as the database stores standardised data across multiple domains in a CDISC compliant format.

Despite these limitations, there are a number of implications from this study. First, and as mentioned earlier, clinically-suspected EVD needs higher priority for treatment and care as there is a substantial mortality associated with this category. Second, large databases such as IDDO need to be better structured and planned right from the start so that it is easy to a) separate baseline variables from follow-up variables during data extraction and b) ensure that important information that might have a bearing on mortality such as vital signs and laboratory investigations at baseline can be easily teased out to enable front-line in-country staff faced with an epidemic/outbreak to access relevant and important data in real-time. Efforts are ongoing in this direction. Third, the extraction of data from the IDDO database was done within a structured operational research training (SORT IT) course, demonstrating once again that this is a useful way of equipping healthcare workers with an understanding about data and implementation research especially during outbreaks and pandemics. ^33^^,^^34^

## Conclusions

In conclusion, during the 2013-2016 EVD outbreak in Guinea, Liberia and Sierra Leone, 14,163 patients were admitted to ETUs and among the 11,274 (80%) patients with outcome recorded, 4,090 (36%) died. Patients with laboratory-confirmed disease had 2.9 times higher risk of death compared to clinically-suspected patients, after adjusting for other co-variables. Clinically-suspected patients nevertheless had a substantial risk of death and more attention needs to be paid to this group in future EVD outbreaks.

## Data availability

### Underlying data

The data that underpin this analysis are available via a governed data access mechanism following review of a data access committee. Data can be requested via the IDDO Ebola Data Platform ( https://www.iddo.org/ebola/data-sharing/accessing-data ). The Data Access Application, Terms of Access and details of the Data Access Committee are available on the website. Briefly, the requirements for access are a request from a qualified researcher working with a legal entity who have a health and/or research remit; a scientifically valid reason for data access which adheres to appropriate ethical principles. The full terms are at: https://www.iddo.org/ebola/data-access-guidelines These data are a part of https://doi.org/10.48688/cpwp-ft84.
