# Supplementary material for: Factors associated with death in patients admitted with Ebola virus disease to Ebola Treatment Units in Guinea, Sierra Leone, and Liberia – December 2013 to March 2016
Source: F1000Res. 2025 Mar 3;13:672. Originally published 2024 Jun 21. [Version 2] doi: 10.12688/f1000research.149612.2 (PMC11880755; doi:10.12688/f1000research.149612.2)
Supplement: Peer review history

# Reviewer report: Reviewer response for version 2

**DOI:** 10.5256/f1000research.178279.r369314

**Published:** 2025-03-04

**Authors:** Michael T Hawkes, Masumbuko Claude Kasereka

**Affiliations:** 1 University of British Columbia, Vancouver, Canada; 2 University of Alberta School of Public Health, Edmonton, Alberta, Canada

We thank the authors for carefully considering our comments and questions!

Is the work clearly and accurately presented and does it cite the current literature?

Yes

If applicable, is the statistical analysis and its interpretation appropriate?

Yes

Are all the source data underlying the results available to ensure full reproducibility?

Yes

Is the study design appropriate and is the work technically sound?

Partly

Are the conclusions drawn adequately supported by the results?

Partly

Are sufficient details of methods and analysis provided to allow replication by others?

Partly

Reviewer Expertise:

Pediatric infectious diseases and global health. Clinical EVD and social resistance to EVD control efforts.

We confirm that we have read this submission and believe that we have an appropriate level of expertise to confirm that it is of an acceptable scientific standard.

# Reviewer report: Reviewer response for version 1

**DOI:** 10.5256/f1000research.164096.r364685

**Published:** 2025-02-08

**Authors:** Aroop Mohanty

**Affiliations:** 1 AIIMS GORAKHPUR, GORAKHPUR, India

At the outset, I express my gratitude to the editorial team to have provided me the opportunity to review this paper in your prestigious journal. Ebola virus causes a very lethal disease and its endemicity in Africa has been found to be very lethal. This paper has been written in clear and simple language and with a proper methodology. The structure of the paper is clear and all the results have been compared very well with the other relevant papers. A bit more can be added on the testing methods deployed to diagnose Ebola as well as a bit more data of recent outbreaks.

Is the work clearly and accurately presented and does it cite the current literature?

Yes

If applicable, is the statistical analysis and its interpretation appropriate?

Yes

Are all the source data underlying the results available to ensure full reproducibility?

Yes

Is the study design appropriate and is the work technically sound?

Yes

Are the conclusions drawn adequately supported by the results?

Yes

Are sufficient details of methods and analysis provided to allow replication by others?

Yes

Reviewer Expertise:

Diagnostic Microbiology

I confirm that I have read this submission and believe that I have an appropriate level of expertise to confirm that it is of an acceptable scientific standard.

# Reviewer report: Reviewer response for version 1

**DOI:** 10.5256/f1000research.164096.r323135

**Published:** 2024-10-09

**Authors:** Michael T Hawkes, Masumbuko Claude Kasereka

**Affiliations:** 1 University of British Columbia, Vancouver, Canada; 2 University of Alberta School of Public Health, Edmonton, Alberta, Canada

This is an analysis of a large multinational database of Ebola virus disease (EVD) patients treated at ETUs in West Africa (2013-2016). The sample size is very large (strength) but the retrospective data are of limited quality (large proportion of missing data), such that biases are likely (weakness). There are many interesting observations that are consistent with past studies. We provide some suggestions to improve the manuscript below:

**Heterogeneity of the “clinically-suspected EVD” group**

 The main comparison is between lab-confirmed and clinically-suspected EVD cases. The “clinically-suspected EVD” group is heterogeneous:

- Negative PCR
- Indeterminate PCR
- EVD positive but low viral load (cycle threshold greater than 36.1)
- No data (missing data)

There are likely cases of malaria, bacterial sepsis, community acquired pneumonia, and other acute infections in this group. There may even be non-infectious pathologies (see comment below on fever). There are likely also some cases of EVD (e.g., when the data were missing). Because cases with positive EVD PCR and low viral load were also included in this group, we may be looking at “mild EVD” in some patients (note that the viral load is a key determinant of mortality, as noted by the authors). It is not unexpected that severe acute non-EVD illness would be associated with some mortality (18.8% in this study). What makes this study difficult to interpret is the heterogeneity of the “clinically-suspected EVD” group. We don’t know what the etiology of their illness was, just that it resembled EVD clinically.

 This is reflected in the study data. In Table 2, known risk factors for EVD transmission (funeral attendance, healthcare worker, contact with suspect case) were present in a higher proportion of lab-confirmed cases than “clinically-suspected EVD” cases. This is consistent with non-EVD etiology in a fraction of “clinically-suspected EVD” cases. For example, an alternative diagnosis of febrile illness, malaria, was found in 35% of “clinically-suspected” cases (versus 19% of laboratory confirmed cases).

**Inclusion criteria ill defined**

 The inclusion criteria (perhaps the same as ETU admission criteria) were not precisely defined for this retrospective database review, and may well be variable between ETUs. For example, one might expect fever to be universal in patients with suspected EVD admitted to an ETU, but it was present in only 77.6% overall and 75.7% of patients with clinically-suspected EVD. Why were afebrile patients admitted to the ETU? (this is actually dangerous as it exposes them to a significant risk of nosocomially-acquired EVD) Inclusion criteria are not clear, which further complicates the interpretation of the cases which were not lab-confirmed.

 Related to the issue of inclusion criteria is the composition of the database itself. The large database is described in some detail. But several questions remain about who was included in this database and who was not included. This question arises, for example, in Table 1, in which the IDDO database had 5448 cases from Guinea while 3814 cases were reported to WHO (143%). One wonders how cases were reported to the IDDO but not the WHO and one questions if there might be duplications or inaccuracies in reporting. We suspect that the WHO records are also imperfect. The absence of any data from the DRC, which experienced the second largest outbreak in history (2018-2020) and has experienced multiple subsequent outbreaks also illustrates that the database (large and multinational) is not truly global and comprehensive. All this calls into question the representativeness of the database and whether the sample (though enormous) may be a selection of EVD patients. The authors are encouraged to provide more granular details on reporting to the IDDO database (mandatory or voluntary) and how the ETUs that contributed data were recruited. We suspect that this is related to the funders (MSF and Wellcome Trust) rather than any scientific sampling strategy. 

**Etiology of illness in the “clinically-suspected EVD” group not examined/investigated**

 Given this limitation (i.e., what exactly are these “clinically-suspected EVD” cases?), it seems reasonable to question and revisit the rationale behind the analysis. In the abstract (Conclusions subsection), and in the concluding paragraph of the article, the authors note that “clinically-suspected patients… still had a substantial risk of death and more attention needs to be paid to this group in future EVD outbreaks.”

 This does indeed seem to justify the present analysis and the conclusion does follow from the data.

 With this rationale in mind, it would seem important to try to identify the etiology of the illness when EVD is clinically suspected but not demonstrated by PCR.

 The authors have noted a high prevalence of malaria which could in part explain the clinical manifestations (although among adults in an endemic area with partial immunity, malaria is not typically lethal). It would be nice to know if the malaria positive patients were treated with an antimalarial, and if mortality was elevated when malaria was present and treatment was not documented.

 For malaria negative patients, bacterial sepsis is on the differential diagnosis. It would be interesting to know how many of these got antibiotics and whether that was associated with differences in mortality.

 A higher proportion of patients with “clinically-suspected EVD” had respiratory symptoms (difficulty breathing, Table 3), suggesting that pneumonia could contribute to mortality in the clinically suspected cases. It would be interesting to know which patients who were EVD negative, malaria-negative, with difficulty breathing, received antibiotics and if this was associated with a difference in mortality.

 Overall, we would would like to see more detailed analysis of the “clinically-suspected EVD” cases and an attempt to classify patients into possible etiologic categories (including mild EVD cases in this groups, as noted above). The large sample size may allow such probing subgroup analyses, which would be helpful to delineate the cause of the illness in the EVD-negative patients. This would go beyond a mere observation of high mortality, toward actionable hypotheses (i.e., treatable etiologies) for improved management of these patients. We don’t think it is satisfactory to identify a high mortality in this subgroup without any attempt to answer why they died. Clinical clues and limited microbiologic data should be leveraged to try to answer this question, recognizing that microbiologic tests were scant and a diagnosis won’t be definitive. After all, this is the difficult task faced by clinicians managing these suspected cases in resource-limited contexts.

**Table 3. Statistical versus clinical significance**

 Despite the heterogeneity of the “clinically-suspected EVD” group, the authors’ analysis could be illuminating for clinicians facing diagnostic uncertainty with febrile patients during an EVD outbreak. One value of this analysis, comparing lab-confirmed and “clinically-suspected EVD,” could be to examine the clinical manifestations in each group to determine which are clinically informative. This is attempted in Table 3, but falls short of its goal.

 We acknowledge the statistically significant differences between lab-confirmed and clinically suspected cases in presenting signs and symptoms (Table 3). Because of the large sample size, these differences all reach statistical significance except for abdominal pain and bleeding (This point is interesting and perhaps unexpected! We would have thought bleeding would be a more specific sign of EVD, a hemorrhagic fever). However, there remains a question about whether the differences are clinically significant.

 Taking, for example, nausea and vomiting (62% in EVD vs 51% in non-EVD, p<0.001), the 11% absolute difference may not be very helpful in distinguishing EVD from other illnesses. The clinical utility (diagnostic accuracy) of clinical exam findings might be better expressed with sensitivity, specificity or likelihood ratios of positive/negative signs (see, for example, the JAMA series on The Rational Clinical Examination). These statistics will likely show that the small differences observed would be of little help in clinical decision-making, even though they are statistically significant. Taking the example of nausea and vomiting as a clinical sign of EVD positivity and using complete cases (ignoring missing data), we calculate a sensitivity of 62%, a specificity of 49%, a likelihood ratio of a positive sign of 1.1 and a likelihood ratio of a negative sign of 0.87. (A good “test” has a positive LR of >10 and/or a negative LR of < 0.1). Thus, nausea and vomiting discriminates poorly between lab-confirmed EVD cases and other illnesses. The same would be true for other clinical signs, even though they are statistically significantly different between the two groups.

 Our interpretation of the findings, therefore, is that the clinical signs are neither sensitive nor specific and are present in a substantial proportion of EVD-negative patients. Thus, the table as presented, with highly statistically significant differences, may mislead all but the most savvy reader knowledgeable in interpreting clinical epidemiology data. Readers will assume that “statistically significant” clinical signs may help identify EVD cases, whereas our interpretation of the findings is quite the opposite: these data show that nothing can replace a PCR.

 To address this limitation, the authors are encouraged to show (for example) sensitivity, specificity, negative and positive likelihood ratios which will make it clear that the clinical signs do not provide actionable information (not clinically informative).

**Table 6**

 “Patients aged 60 years and above had a significantly higher risk of death compared to that in ≤5-year-old children.”

 The authors go on to note in the discussion that old age is a risk factor for mortality and cite other studies in support of this finding.

 The data do indeed show this, but the conclusion misses another major, important finding. The RR of death is significantly lower in the 6-18 year olds and lower in the 19-39 year olds relative to the reference group (<=5 years). Thus, the “U-shaped” mortality curve, with higher mortality at the extremes of age should be the “take-away” observation from the data, not just elevated mortality in the elderly. We request that the authors amend this sentence to include elevated mortality in children and infants under 5 years of age (relative to older children and young adults) as well as elevated mortality in the elderly.

**Discussion -**

 We were perplexed by the following statement and citation in the Discussion: “Oral cephalosporins may reduce the case fatality…” (citation Gignoux E, Azman AS, de Smet M , et al.: Effect of Artesunate-Amodiaquine on Mortality Related to Ebola Virus Disease. N. Engl. J. Med. 2016 Jan 7 [cited 2023 Jul 15]; 374(1): 23–32.) The citation compares two artemisinin combination therapy (ACT) regimens and does not examine oral cephalosporins. Broad-spectrum intravenous (not oral) cephalosporins would seem to be indicated in critically ill EVD patients in whom a blood culture is not available (Plachouras D, Monnet DL, Catchpole M. Severe Ebola virus infection complicated by gram-negative septicemia. N Engl J Med. 2015 Apr 2;372(14):1376-7.).

 We request that the authors delete or modify this sentence and citation.

**Missing data**

 The authors are to be congratulated on reporting the missing data transparently in the Tables. Missing data is acknowledged as a limitation in the Discussion. Nonetheless, this remains a major limitation which threatens the validity of the findings.

 When, for example, the chi-squared statistic was calculated (e.g. ,in Table 2), were the missing cases disregarded? Or was missing included as a category in a “three-level” categorical variable (yes/no/missing) in the contingency table? The “complete case” analysis (ignoring missing data) is actually a “naïve” approach that is prone to biases when the data are not missing at random. It would have been nice to see attempts to address data missingness through statistical analyses (e.g., multiple imputation).

Is the work clearly and accurately presented and does it cite the current literature?

Yes

If applicable, is the statistical analysis and its interpretation appropriate?

Yes

Are all the source data underlying the results available to ensure full reproducibility?

Yes

Is the study design appropriate and is the work technically sound?

Partly

Are the conclusions drawn adequately supported by the results?

Partly

Are sufficient details of methods and analysis provided to allow replication by others?

Partly

Reviewer Expertise:

Pediatric infectious diseases and global health. Clinical EVD and social resistance to EVD control efforts.

We confirm that we have read this submission and believe that we have an appropriate level of expertise to confirm that it is of an acceptable scientific standard, however we have significant reservations, as outlined above.

# Response

**Published:** 2025-02-14

**Authors:** Trokon Yeabah

**Affiliations:** Division of Infectious Disease, National Public Health Institute of Liberia, Monrovia, Montserrado, Liberia

Response to Reviewer 1

 7 Views

*09 Oct 2024 | for Version 1*

 Michael T Hawkes, University of British Columbia, Vancouver, Canada 

 Masumbuko Claude Kasereka, University of Alberta School of Public Health, Edmonton, Alberta, Canada 

*format_quote* Cite this report *speaker_notes* Responses(0)

Approved With Reservations

**Response**:

 We thank the reviewer for his excellent and in-depth review and the time and energy spent on his review. We have responded to the reviewer’s comments below in a point-by-point fashion, but do point out that we have been unable to address some of them. For this, we apologise. We have made some revisions to the paper using tracking changes so these can easily be found. 

Heterogeneity of the “clinically-suspected EVD” group 

**Response:**

 We have tried hard in the IDDO database to get more data as suggested on the clinically-suspected EVD group such as who had malaria, sepsis, other acute infections and so on. It is a very complicated and non-user-friendly database and we have been unable to obtain these data. We are sorry about this, as it was an excellent suggestion.

Inclusion criteria ill-defined

**Response:**

 We have better explained the inclusion criteria under Study population. We have stated in lines 191- 193: *“The study population included all patients classified as having either clinically-suspected or laboratory-confirmed EVD who were admitted at the 22 ETUs in Guinea, Liberia and Sierra Leone between December 2013 and March 2016 and who were captured in the IDDO database.”* Unfortunately, we do not know how the decisions about admission were actually made at the ETUs as these data were not included or entered to the IDDO database.

 We also do not know the reasons for the discrepancy in 5448 EVD cases from Guinea in the IDDO database and the 3814 cases from Guinea reported to WHO. We suspect that cases reported to WHO included just those with confirmed EVD and those with suspected EVD were not reported, but this is speculation on our part. As explained under Study Population, our cases were those captured in the IDDO database and we do not know if these numbers are fully representative of the whole. We have commented on all this as a limitation in the Discussion in lines 378-382.

 We did not report on cases from DRC as the project was just looking at cases from Guinea, Sierra Leone and Liberia.

Aetiology of illness in the “clinically-suspected EVD” group not examined/investigated

**Response:**

 The suggestion to deep-dive into the data to try and verify the aetiology of the illness when EVD is clinically suspected but not demonstrated by PCR is excellent. As explained earlier, we apologise but have been unable to do this due to the complexity of the IDDO database and inconsistent documentation.

Table 3. Statistical versus clinical significance

**Response:**

 Thank you for this suggestion. We accept that the large numbers make most of the comparisons statistically significant. However, we feel that Table 3 should remain as an important part of the data. We also feel that an additional Table(s) showing sensitivity, specificity, negative and positive likelihood ratios will add to the length of the paper and might make it more complicated for the reader to understand. We have therefore not gone ahead with the suggestion. 

Table 6

**Response:**

 Thank you and good point. We have amended the sentence about higher risk of death in certain age groups as follows in lines 316-317: *“Patients aged 60 years and above had a significantly higher risk of death compared to that in ≤5-year-old children, whereas those aged 6-39 years had a significantly lower risk of death compared with children aged 5 years or younger.”*

Discussion and references

**Response:**

 Thank you for highlighting the confusion about the referencing with cephalosporins and antimalarial treatment. We had made mistakes here which we have corrected. There were also two duplications in the references and we have now amended these and the references are in order. 

Missing data

**Response:**

 Thank you for the positive comment. We have not conducted a ‘complete case’ analysis. We have included missing data as part of all analyses, and this can be clearly seen in Table 6 where we have included “unknown” as a subcategory in all the variables included. In the other descriptive tables showing univariate analysis, we have calculated the percentages after exclusion of missing data but have included the missing data when calculating the chi square test. We hope this clarifies. Multiple imputation analysis is not recommended when the missing data are not missing at random, meaning the reason for missing data is related to the missing value itself, potentially introducing bias in the analysis. Since we do not know if the missing data are missing at random, we think it is prudent not to attempt a multiple imputation analysis. We do mention missing data as an important limitation of the study.
